# Supplementary material for: Functionally redundant roles of ID family proteins in spermatogonial stem cells
Source: Stem Cell Reports. 2024 Sep 26;19(10):1379–88. doi: 10.1016/j.stemcr.2024.08.011 (PMC11561458; doi:10.1016/j.stemcr.2024.08.011)
Supplement: Document S1. Figures S1 and S2 and supplemental experimental procedures [file mmc1.pdf]

Stem Cell Reports, Volume *19*

Supplemental Information

Functionally redundant roles of ID family proteins in spermatogonial stem cells

Hue M. La, Ai-Leen Chan, Ashlee M. Hutchinson, Bianka Y.M. Su, Fernando J. Rossello, Ralf B. Schittenhelm, and Robin M. Hobbs

SUPPLEMENTAL INFORMATION

Functionally Redundant Roles of ID Family Proteins in Spermatogonial Stem Cells

Hue M. La, Ai-Leen Chan, Ashlee M. Hutchinson, Bianka Y.M. Su, Fernando J. Rossello, Ralf B. Schittenhelm and Robin M. Hobbs

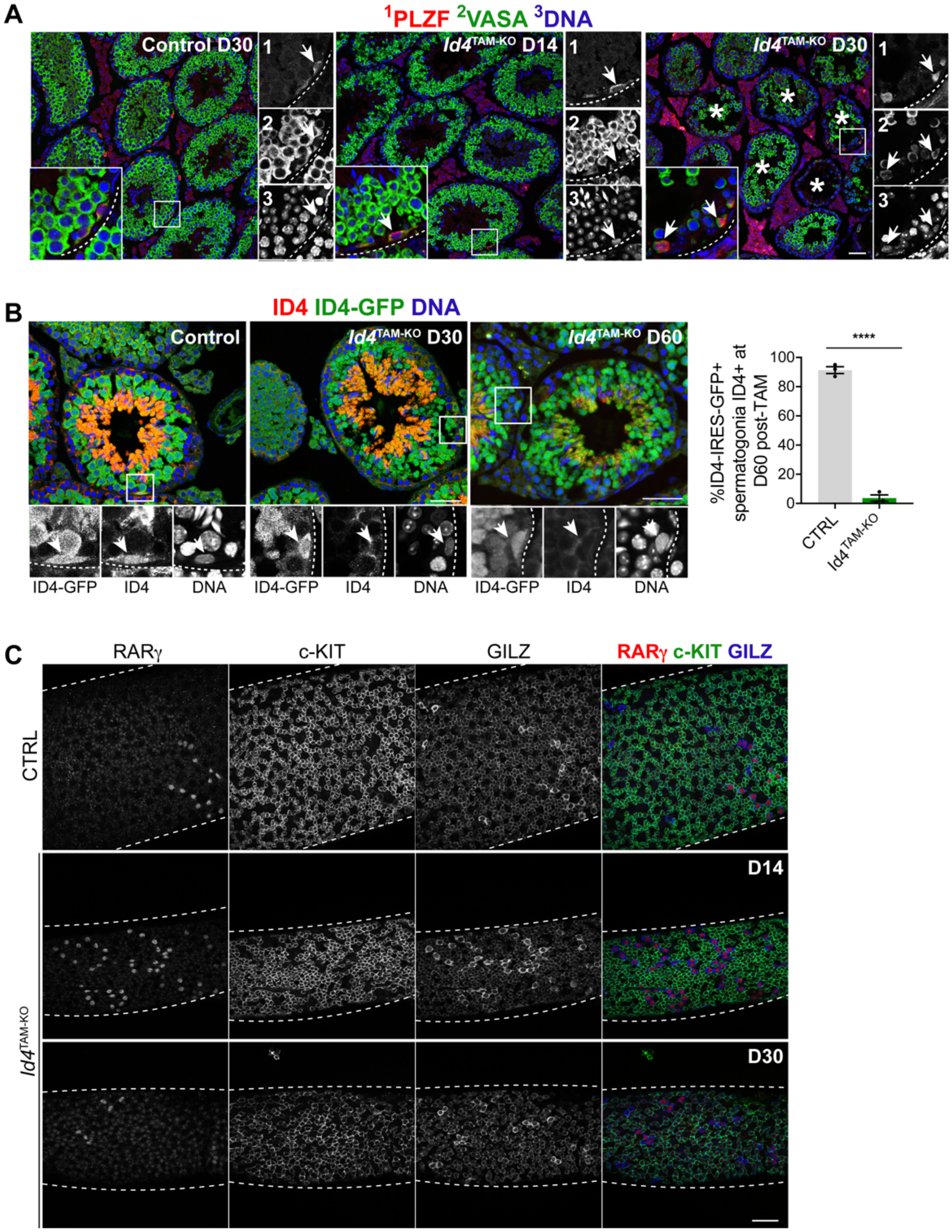

Figure S1. Effects of *Id4* deletion on the adult male germline, related to Figure 2

(A, B) Representative IF of testis sections from $Id4^{flox/flox}$ UBC-CreER ($Id4^{TAM\text{-}KO}$) and Cre-negative control mice treated with TAM and harvested at the indicated time points. Arrows indicate PLZF+ (A) and Id4-GFP+ (B) spermatogonia. Insets show higher magnification details of indicated areas. Asterisks: degenerating tubules. Graph in B shows percentage of GFP+ spermatogonia that are ID4+ D60 post-TAM as an indicator of *Id4* deletion. Data are mean±SEM (*n*=3 mice per genotype).

(C) Representative wholemount IF of seminiferous tubules from $Id4^{TAM\text{-}KO}$ and control adult mice post-TAM.

Scale bars, 50 μm. Dashed lines indicate tubule basement membrane or tubule profile. Significance by two-tailed Student's t-test (*****P*<0.0001).

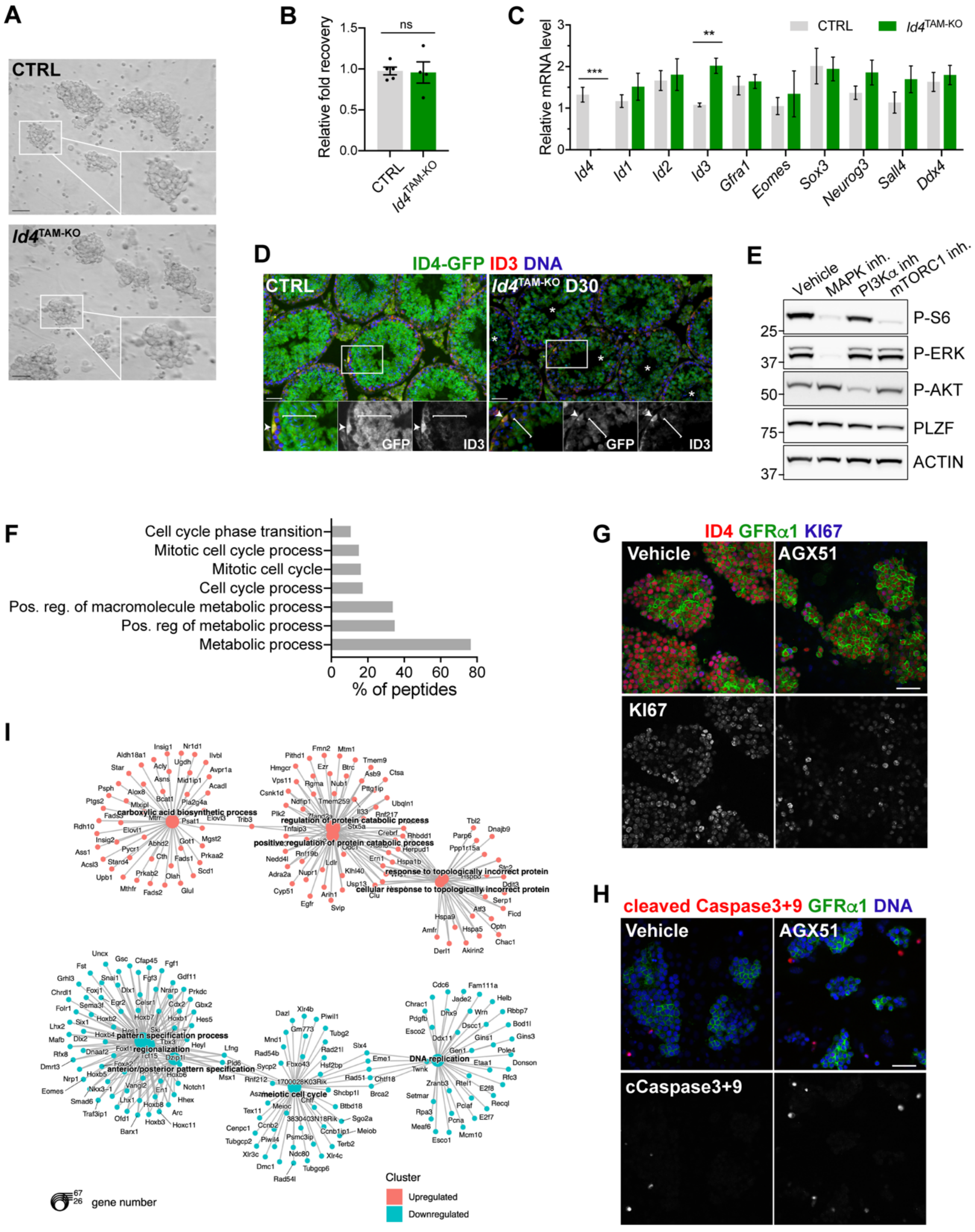

Figure S2. Disruption of *Id* family function in A$_{undiff}$, related to Figure 3

(A) Phase-contrast light microscope images of A$_{undiff}$ cultures generated from *Id4*$^{TAM\text{-}KO}$ and control mice D14 after TAM. Insets show higher magnification details of colonies.

(B) Cultures of A$_{undiff}$ from A were plated on 12-well plates (0.25x10^6 cells/well) and expanded for 2 weeks prior to harvest and counting. Graph indicates relative fold cell recovery (*n*=5 control and *n*=4 *Id4*$^{TAM\text{-}KO}$ independent cultures from separate mice).

(C) RT-qPCR analysis of A$_{undiff}$ cultures from A. Expression levels are corrected to b-actin and normalized to control sample. Significant changes in expression are indicated.

(D) Representative IF of testis sections from control and *Id4*$^{TAM-KO}$ mice D30 post-TAM (n=4 mice per genotype). Insets show higher magnification details. Asterisks indicate tubules with spermatocyte depletion. Brackets in insets indicate spermatocyte and spermatid populations. Arrows: GFP+ ID3+ spermatogonia. Note that GFP expression from the *Id4*$^{IRES-GFP}$ reporter is maintained following deletion of floxed *Id4* exons although levels are reduced compared to the unrecombined knock-in allele.
(E) Representative western blot of cultured wildtype A_{undiff} treated for 3h with inhibitors to the indicated signalling pathways (*n*=3 independent cultures from separate mice).
(F) Gene ontology analysis of ID4 interacting proteins identified from IP and mass spectrometry analysis of Fig. 3F.
(G, H) Representative IF analysis of wildtype A_{undiff} cultures treated with vehicle or pan-ID inhibitor AGX51 for 20h. Grayscale images of indicated channels are shown.
(I) Pathway analysis of differentially expressed genes identified from RNA-seq of wildtype A_{undiff} cultures treated with vehicle or AGX51 as in Fig. 3J.
Mean values ± SEM are shown in B and C. Scale bars, 50μm. Significance by two-tailed Student's t-test (P>0.05 (ns), **P<0.01, ***P<0.001).

Table S1. Identification of ID4 interacting proteins in cultured A_{undiff}, related to Figure 3 (Excel file).

Proteins interacting with ID4 in independent cultures of wildtype A_{undiff} were identified by ID4 IP and high-resolution mass spectrometry analysis as in Figure 3F. The table includes interaction partners identified in at least 2 out of 3 IPs versus non-specific IgG controls. Numbers of MS2 spectra are indicated. Proteins detected in respective IgG control IPs were excluded (-).

Table S2. Analysis of gene expression in cultured A_{undiff} following ID inhibition, related to Figure 3 (Excel file).

Cultured A_{undiff} were treated with vehicle or pan-ID inhibitor AGX51 for 20h and then analysed by RNA-Seq (n=4 independent cultures from separate mice). Table shows differentially expressed genes (FDR <0.05 and fold change >1.5).

SUPPLEMENTAL EXPERIMENTAL PROCEDURES

Mouse models and treatment

Wildtype adult mice were 6-8 weeks old and C57BL/6J background. *Id4*$^{\text{IRES-GFP}}$ mice were maintained on a mixed FVBN/CBA/C57BL6 background and are described elsewhere (Best et al., 2014). Mice homozygous for the *Id4*$^{\text{IRES-GFP}}$ allele were used for GFP expression analysis. *Id4*$^{\text{TAM-KO}}$ mice were generated by crossing *Id4*$^{\text{IRES-GFP}}$ mice with the UBC-CreER strain (Jackson Laboratories) (Chan et al., 2017; Ruzankina et al., 2007). The IRES-GFP reporter is retained following Cre-dependent deletion of floxed *Id4* exons (Best et al., 2014). Homozygous *Id4*$^{\text{IRES-GFP}}$; hemizygous UBC-CreER mice were used as experimental mice and homozygous littermate *Id4*$^{\text{IRES-GFP}}$ mice without UBC-CreER as controls. To induce *Id4* deletion, 6–8-week-old adult *Id4*$^{\text{TAM-KO}}$ mice were injected with 2 mg TAM (Sigma) daily for 2 consecutive days as described (Chan *et al.*, 2017). Animal studies were performed in accordance with the Australian Code of Practice for the Care and Use of Animals for Scientific Purposes. Experiments were subject to approval by Monash University and Medical Centre Animal Ethics Committees.

Immunofluorescence

For sections, testes were fixed with 4% paraformaldehyde (PFA) in phosphate-buffered saline (PBS) overnight at 4^0C, cryoprotected with 30% sucrose and embedded in OCT (Tissue-Tek) for sectioning. Sections were blocked in 10% FBS (GE Healthcare) with 2% bovine serum albumin (BSA) (Sigma) in PBS, then incubated at 4^0C overnight with primary antibodies in blocking solution. After PBS washes, sections were incubated with Alexa Fluor-conjugated secondary antibodies raised in Donkey (Thermo) and DAPI. For wholemount analysis, testes were detunicated, seminiferous tubules teased apart and rinsed in PBS, then fixed in 4% PFA for 5 hours

at 4^0C. Fixed tubules were washed in PBS prior to blocking in 0.3% Triton X-100 in PBS (PBSX) supplemented with 10% FBS and 2% BSA. Tubules were incubated with primary antibodies in PBSX with 1% BSA at 4^0C overnight. Samples were washed in PBSX, and primary antibodies detected as above. For IF analysis of cultured A_{undiff}, cells grown on Lab-Tek Chamber slides were fixed in 4% PFA and processed as described (Chan *et al.*, 2017). Slides were mounted in Vectashield mounting medium (Vector Labs) and imaged with Zeiss LSM780 FCS and Nikon C1 confocal microscopes at the Monash Micro Imaging (MMI) platform. ImageJ was used for image processing. Whole-mount tubules were assigned to different stages of the seminiferous epithelium cycle based on abundance and morphology of differentiating and undifferentiated spermatogonial populations as described previously (Chan *et al.*, 2017). Primary antibodies were as follows: rabbit anti-ID4 (clone 82-12, 1:2000) and rabbit anti-ID3 (clone 6-1, 1:2000) (CalBioreagents); chicken anti-GFP (ab13970, 1:5000), rabbit anti-SALL4 (ab29112, 1:2000) and rabbit anti-SYCP3 (ab15093, 1:500) (Abcam); goat anti-PLZF (AF2944, 1:500), anti-GFRα1 (AF560, 1:250), anti-SOX3 (AF2569, 1:250), anti-c-KIT (AF1356, 1:250), anti-E-cadherin (AF748, 1:250) and rabbit anti-EOMES (clone 1219A, 1:1000) (R&D Systems); rat anti-KI67 (clone SolA15, 1:250) and anti-GILZ (clone CFMKG15, 1:1000) (Thermo); rabbit anti-VASA (clone D10C5, 1:500), anti-RARγ (clone D3A4, 1:500), anti-cleaved caspase 3 (#9579, 1:500), and anti-cleaved caspase 9 (#9509, 1:250) (Cell Signaling Technology).

Flow cytometry

Single cell suspensions were generated from testis by digestion with type II collagenase (Sigma) (La et al., 2018a). Cells were stained for 25 minutes on ice with antibodies in phosphate-buffered saline (PBS) with 2% fetal bovine serum (FBS). Antibodies were as follows: PE anti-E-cadherin

clone DECMA-1 (1:250), APC anti-c-KIT clone 2B8 (1:500), PE-Cy7 anti-CD49f (integrin α6) clone GoH3 (1:500) (Thermo and BioLegend). DAPI was used for live/dead cell discrimination. Cell cycle analysis was performed with a Click-iT EdU Pacific Blue Flow Cytometry Kit (Thermo) according to manufacturer's instructions and 3-hour 10μM EdU treatment (Legrand et al., 2019). Analysis of fixed and permeabilised A_{undiff} for PLZF and EOMES is previously described (La et al., 2018b). Cells were sorted and analysed at Monash FlowCore using an Influx Cell Sorter and LSR Fortessa X-20 (BD Biosciences). Data were processed with FlowJo software.

Cell culture and treatment

A_{undiff} cultures were generated and maintained on mitotically-inactivated mouse embryonic fibroblasts (MEF) in StemPro-34 media supplemented with 10ng/ml GDNF, 10ng/ml bFGF, 20ng/ml EGF, 25μg/ml insulin and other additives as described (La *et al.*, 2018a). To establish cultures, A_{undiff} were enriched from testis cell suspensions using biotinylated anti-CD9 antibody clone MZ3 (BioLegend, 1:400) and EasySep Biotin Positive Selection kits (Stem Cell Technologies). For inhibitor treatment, adherent feeder cells were depleted from harvested cells on tissue culture plates for 1-2 hours and non-adherent spermatogonia collected and plated onto 12-well plates coated with Geltrex matrix (Thermo) as detailed (La et al., 2022). Inhibitors (MedChemExpress and Selleckchem) were dissolved in DMSO and diluted in supplemented StemPro-34 media to the following concentrations: 5μM PD0325901 (MAPK inhibitor), 1μM alpelisib (PI3Kα), 20nM rapamycin (mTORC1) and 80μM AGX51 (pan-ID). Media for inhibitor studies contained GDNF, bFGF, EGF, insulin and other supplements as above.

Immunoprecipitation and western blotting

Immunoprecipitation and western blotting were performed as described (La *et al.*, 2018a; La *et al.*, 2022). The following antibodies were used for western blotting: rabbit anti-ID4 (clone 82-12, 1:1000) and anti-ID3 (clone 6-1, 1:1000) (CalBioreagents); rabbit anti-TCF3 (PA5-78190, 1:1000) (Thermo); hamster anti-PLZF (clone 9E12) (Hobbs et al., 2010); rabbit anti-phospho-RPS6 (Ser235/236) (clone D57.2.2E, 1:2000), anti-phospho-p44/42 MAPK (Thr202/Tyr204) (clone D13.14.4E, 1:2000) and anti-phospho-AKT (Ser473) (D9E, 1:2000) (Cell Signaling Technology); and mouse anti-β-ACTIN (Sigma, 1:2000). Band intensity was quantified using ImageJ. Immunoprecipitation with rabbit anti-TCF3 antibody (PA5-78190, Thermo) was performed as detailed below.

Mass spectrometry

ID4 complexes were immunoprecipitated from wildtype cultured A_{undiff} lysate with Dynabeads coupled with rabbit anti-ID4 antibody (clone 82-12, CalBioreagents) using a Dynabeads Antibody Coupling Kit (Thermo Fisher) as described (La *et al.*, 2018a; Legrand *et al.*, 2019). Dynabeads coupled to non-specific rabbit IgG were used as control. Proteins were eluted in 0.2 M glycine pH 2.5 and analysed by the Monash Proteomics & Metabolomics Platform. Immunoprecipitated proteins were reduced with 10 mM TCEP (Thermo), alkylated with 40 mM chloroacetamide (Sigma Aldrich), and digested with sequencing grade trypsin (Promega). Samples were acidified with 1% formic acid (FA) and purified using OMIX C18 Mini-Bed tips (Agilent). Tryptic peptides were separated using a Dionex UltiMate 3000 RSLCnano system and analysed with an Orbitrap Fusion Tribrid mass spectrometer (Thermo). Raw data files were analysed with the Byonic software suite (ProteinMetrics) using a mouse protein sequence database (Uniprot/SwissProt).

RNA-Sequencing

Quality of RNA extracted from A_{undiff} as above was assessed using a Bioanalyzer and RNA quantity by Qubit. RNA-Seq was performed using a custom in-house multiplex method (Grubman et al., 2021). Index was added during initial pA priming and pooled samples amplified using a template switching oligo. P5 was added by tagmentation by Nextera transposase and PCR. Pooled libraries were sequenced on a NextSeq2000 by Monash Health Translation Precinct (MHTP) Medical Genomics Facility. Data were demultiplexed and processed by the Monash Bioinformatics Platform using the RNAsik pipeline. Raw counts files were uploaded to the Degust Webapp (http://degust.erc.monash.edu), which uses limma-voom for statistical analysis. Cut-off for differentially expressed genes was false discovery rate (FDR) <0.05 and fold change >1.5. ComplexHeatmap and clusterProfiler tools (Bioconductor) were used to generate heatmaps and for pathway analysis (Wu et al., 2021).

SUPPLEMENTAL REFERENCES

Hobbs, R.M., Seandel, M., Falciatori, I., Rafii, S., and Pandolfi, P.P. (2010). Plzf regulates germline progenitor self-renewal by opposing mTORC1. Cell *142*, 468-479.

Ruzankina, Y., Pinzon-Guzman, C., Asare, A., Ong, T., Pontano, L., Cotsarelis, G., Zediak, V.P., Velez, M., Bhandoola, A., and Brown, E.J. (2007). Deletion of the developmentally essential gene ATR in adult mice leads to age-related phenotypes and stem cell loss. Cell Stem Cell *1*, 113-126.

Wu, T., Hu, E., Xu, S., Chen, M., Guo, P., Dai, Z., Feng, T., Zhou, L., Tang, W., Zhan, L., et al. (2021). clusterProfiler 4.0: A universal enrichment tool for interpreting omics data. Innovation (Camb) *2*, 100141.
