# Supplementary material for: Functionally redundant roles of ID family proteins in spermatogonial stem cells
Source: Stem Cell Reports. 2024 Sep 26;19(10):1379–88. doi: 10.1016/j.stemcr.2024.08.011 (PMC11561458; doi:10.1016/j.stemcr.2024.08.011)
Supplement: Document S2. Article plus supplemental information [file mmc4.pdf]

Stem Cell Reports
Report
OPEN ACCESS

Functionally redundant roles of ID family proteins in spermatogonial stem cells

Hue M. La,[1,2,3,4] Ai-Leen Chan,[1,2] Ashlee M. Hutchinson,[1,2] Bianka Y.M. Su,[1,2] Fernando J. Rossello,[4,5,6,7] Ralf B. Schittenhelm,[8] and Robin M. Hobbs[1,2,9,*]

[1]Centre for Reproductive Health, Hudson Institute of Medical Research, Melbourne, VIC 3168, Australia
[2]Department of Molecular and Translational Sciences, Monash University, Melbourne, VIC 3800, Australia
[3]University of Melbourne Centre for Cancer Research, University of Melbourne, Melbourne, VIC 3000, Australia
[4]Department of Clinical Pathology, University of Melbourne, Melbourne, VIC 3000, Australia
[5]Murdoch Children's Research Institute, The Royal Children's Hospital, Melbourne, VIC 3052, Australia
[6]Novo Nordisk Foundation Center for Stem Cell Medicine, Murdoch Children's Research Institute, Melbourne, VIC 3052, Australia
[7]Australian Regenerative Medicine Institute, Monash University, Melbourne, VIC 3800, Australia
[8]Monash Proteomics & Metabolomics Platform, Monash Biomedicine Discovery Institute & Department of Biochemistry and Molecular Biology, Monash University, Clayton, VIC 3800, Australia
[9]Lead contact
*Correspondence: robin.hobbs@monash.edu
https://doi.org/10.1016/j.stemcr.2024.08.011

SUMMARY

Spermatogonial stem cells (SSCs) are essential for sustained sperm production, but SSC regulatory mechanisms and markers remain poorly defined. Studies have suggested that the *Id* family transcriptional regulator *Id4* is expressed in SSCs and involved in SSC maintenance. Here, we used reporter and knockout models to define the expression and function of *Id4* in the adult male germline. Within the spermatogonial pool, *Id4* reporter expression and inhibitor of DNA-binding 4 (ID4) protein are found throughout the GFRα1+ fraction, comprising the self-renewing population. However, *Id4* deletion is tolerated by adult SSCs while revealing roles in meiotic spermatocytes. Cultures of undifferentiated spermatogonia could be established following *Id4* deletion. Importantly, ID4 loss in undifferentiated spermatogonia triggers ID3 upregulation, and both ID proteins associate with transcription factor partner TCF3 in wild-type cells. Combined inhibition of IDs in cultured spermatogonia disrupts the stem cell state and blocks proliferation. Our data therefore demonstrate critical but functionally redundant roles of IDs in SSC function.

INTRODUCTION

Inhibitor of DNA-binding (ID) proteins play roles in cell development, proliferation, and fate and are dysregulated in neurological disorders and cancer (Wang and Baker, 2015). Mammals possess four members (ID1–ID4), containing a helix-loop-helix (HLH) motif mediating dimerization with other HLH proteins but lack DNA-binding domains. IDs heterodimerize with basic-HLH (bHLH) transcription factors, including E-proteins and class II factors (Wang and Baker, 2015). Heterodimers of E-proteins and class II factors bind E-box sequences to regulate gene expression. However, ID-bHLH heterodimers cannot bind DNA and are transcriptionally inactive. Since bHLH factors regulate genes associated with differentiation and cell-cycle arrest, IDs maintain cells in an undifferentiated and proliferative state (Roschger and Cabrele, 2017). ID function is also mediated through interaction with non-bHLH proteins (Roschger and Cabrele, 2017). Studies of *Id* knockout models have indicated that IDs have both redundant and unique roles (Lyden et al., 1999; Wang and Baker, 2015).

Sustained sperm production is dependent on spermatogonial stem cells (SSCs) in the testis seminiferous epithelium (La and Hobbs, 2019). SSCs self-renew and generate differentiation-destined progenitors that produce gametes through spermatogenesis. SSCs are contained within a heterogeneous pool of undifferentiated spermatogonia (type A undifferentiated/A_{undiff}) (Figure 1A). A_{undiff} exist as single (A_s) and chains of 2 (A_{pr}) or more cells (A_{al}) formed due to incomplete cytokinesis. A_{undiff} expressing receptor GFRα1, the majority of A_s and A_{pr}, and a minor subset of A_{al}, comprise the SSC pool in which cells interconvert between renewing- and differentiation-biased states. EOMES, PDX1, and PLVAP mark the most undifferentiated or primitive cells (La et al., 2018b; Nakagawa et al., 2021). A_{undiff} positive for SOX3, RARγ, and NGN3, which are mostly A_{al} plus some A_s and A_{pr}, act as progenitors although can revert to SSCs, particularly under regenerative conditions (La and Hobbs, 2019; Nakagawa et al., 2010). PLZF, SALL4, and E-Cadherin are expressed throughout the A_{undiff} pool and early differentiation stages (Figure 1A) (La and Hobbs, 2019; La et al., 2018b). In response to retinoic acid, progenitors induce c-KIT and differentiate, then undergo multiple divisions before generating meiotic spermatocytes.

SSC self-renewal depends on growth factors from testis somatic cells including glial cell-derived neurotrophic factor (GDNF) (Yoshida, 2019). *Id4* was identified in a screen for GDNF-induced genes in cultured A_{undiff}, and *Id4* knockdown inhibited SSC expansion *in vitro* (Oatley et al., 2011).

Published by Elsevier Inc. on behalf of International Society for Stem Cell Research.
This is an open access article under the CC BY license (http://creativecommons.org/licenses/by/4.0/).

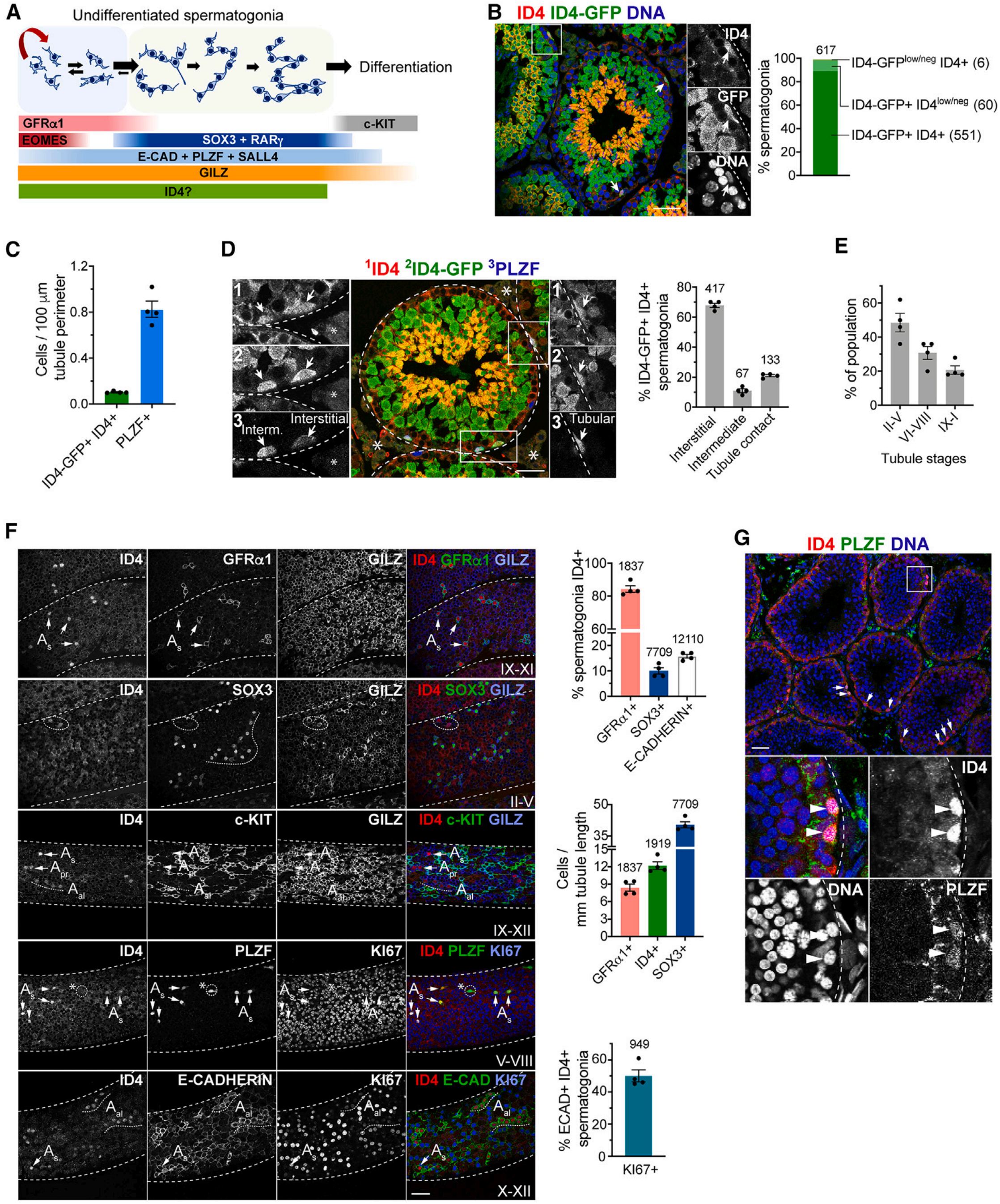

Figure 1. Expression pattern of *Id4* in adult testis

(A) Spermatogonial hierarchy and markers associated with A_{undiff} populations.

(B) Representative IF of adult *Id4*$^{IRES\text{-}GFP}$ testis sections. Graph shows percentage overlap between GFP and ID4 expression (n= 4 mice). Arrows: GFP+ ID4+ spermatogonia.

(C) Abundance of GFP+ ID4+ and PLZF+ spermatogonia in sections from B normalized to tubule perimeter.

(D) Representative IF of adult *Id4*$^{IRES\text{-}GFP}$ testis sections showing localization of GFP+ ID4+ spermatogonia within tubules. Arrows: GFP+ ID4+ spermatogonia. Graph shows percentage of cells localized at distinct tubule regions (n = 4 mice). Numbered panels show grayscale images of the indicated markers. Asterisks: autofluorescent interstitial regions.

(E) Distribution of GFP+ ID4+ spermatogonia to the indicated ranges of seminiferous tubule stages from D. 60 tubule cross-sections scored per animal (n = 4 mice).

(legend continued on next page)

Id4 null male mice exhibit age-dependent germline degeneration while overexpression blocks SSC-to-progenitor transition, supporting a role for ID4 in SSC maintenance (Helsel et al., 2017; Oatley et al., 2011). Expression of an Id4-GFP transgene was prominent in a subset of A_s and transplantation capacity strongly enriched within the Id4-GFP+ population, suggesting that SSC activity is primarily restricted to *Id4*-expressing A_s (Chan et al., 2014; Helsel et al., 2017). However, single-cell RNA sequencing (scRNA-seq) analysis and quantitative reverse-transcription PCR (RT-qPCR) of A_{undiff} fractions suggested broad expression of *Id4* within A_{undiff} (Hermann et al., 2018; Kitadate et al., 2019; La et al., 2018b). The identity of ID4+ cells in the germline therefore remains ambiguous. Moreover, ID4 targets in SSCs are poorly defined.

Here, using a knockin reporter, we characterize *Id4* expression in the male germline and study effects of *Id4* loss on SSCs. We find that *Id4* expression overlaps with that of GFRα1 and SSC function remains intact following *Id4* deletion. We provide evidence that ID4 loss in A_{undiff} is compensated by ID3, and combined ID inhibition disrupts the SSC state. Our data resolve discrepancies concerning the identity of ID4+ spermatogonia and support a redundant role for IDs in SSC maintenance.

RESULTS AND DISCUSSION

Characterization of ID4+ cells in the adult male germline

Id4 expression in the germline has been inferred from a high-copy reporter, but studies have conflicted with scRNA-seq and other data (Chan et al., 2014; Kitadate et al., 2019; La et al., 2018b). The correlation between *Id4* transcription and protein in the germline also remains unclear. We therefore analyzed an $Id4^{IRES\text{-}GFP}$ knockin mouse that provides readout of endogenous gene activity (Best et al., 2014). Adult testis sections were analyzed by immunofluorescence (IF) for GFP and ID4 to compare gene activity with protein. GFP and ID4 were detected in a subset of spermatogonia in the seminiferous epithelium basal layer (Figure 1B). GFP was also detected in spermatocytes and spermatids toward the lumen, indicating that *Id4* is expressed during spermatogenesis although little specific nuclear ID4 signal was evident in spermatocytes (Figure 1B).

Extensive overlap between GFP and ID4 in spermatogonia (~90%) (Figure 1B) indicated a high degree of correlation between gene activity and protein and confirmed ID4 antibody specificity. GFP+/ID4+ spermatogonia were found exclusively within the PLZF+ population although represented a small fraction (~10%) of PLZF+ cells (Figures 1C and 1D). Most GFP+/ID4+ spermatogonia (~70%) were in proximity to the interstitium with minor fractions at regions of tubule-tubule contact or intermediate areas (Figure 1D), consistent with biased SSC localization (Hara et al., 2014; Kitadate et al., 2019). GFP/ID4+ spermatogonia were present throughout the seminiferous epithelium cycle, suggesting they may be SSCs, although were most evident at early stages (II–V) (Figure 1E) (Nakagawa et al., 2021; Tegelenbosch and de Rooij, 1993).

To characterize ID4+ spermatogonia, we analyzed wild-type seminiferous tubules by whole-mount IF (Figure 1F). In agreement with previous analysis (Kitadate et al., 2019), ID4 was mostly restricted to the GFRα1+ population and the majority of GFRα1+ cells were ID4+ (~80%). A small proportion of SOX3+ progenitors were ID4+ (~10%) but ID4+ cells were not found in the c-KIT+ differentiating population. ID4+ spermatogonia were as abundant as GFRα1+ cells but much less than SOX3+ cells (Figure 1F). While predominantly in A_s and A_{pr}, ID4 was detected in some E-Cadherin+ A_{al} (Figure 1F), consistent with previous reports (Kitadate et al., 2019; Sharma et al., 2019). ~50% of E-Cadherin+/ID4+ spermatogonia were KI67+, confirming mitotic activity (Figure 1F). Nuclear ID4 was also evident in PLZF+ spermatogonia of the marmoset, suggesting conservation of function (Figure 1G). Although more broadly expressed than previously suggested (Chan et al., 2014), our data illustrate that *Id4* expression is mainly restricted to GFRα1+ cells, indicating a functional role in SSCs.

Effects of *Id4* deletion on adult SSCs

Id4 null mice survive into adulthood but males exhibit germline loss, suggesting defects in SSC maintenance and/or development (Oatley et al., 2011). To define the role of ID4 in SSCs, we developed an inducible knockout model ($Id4^{TAM\text{-}KO}$) by crossing $Id4^{IRES\text{-}GFP}$ mice, containing *loxP* sites flanking exons 1 and 2 (Best et al., 2014), with UBC-CreER transgenic mice, allowing tamoxifen (TAM)-induced gene deletion in spermatogonia (Chan et al., 2017).

(F) Representative whole-mount IF of adult wild-type seminiferous tubules. Tubule stage is indicated. Selected A_{undiff} are highlighted with arrows. Graphs show the percentage of spermatogonial populations expressing ID4 (top), abundance of spermatogonial populations (middle), and percentage of ID4+ E-Cadherin+ spermatogonia KI67+ (bottom). 37–40 mm of tubules analyzed on one tubule face per animal (*n* = 4 mice).
(G) Representative IF of adult marmoset testis sections (*n* = 2 animals). Arrows: ID4+ PLZF+ spermatogonia. Graphs show mean ± SEM. Total numbers of scored cells are indicted. Tubule basement membrane or profile is indicated with dashed lines. Insets show higher magnification details. Scale bars 50 μm.

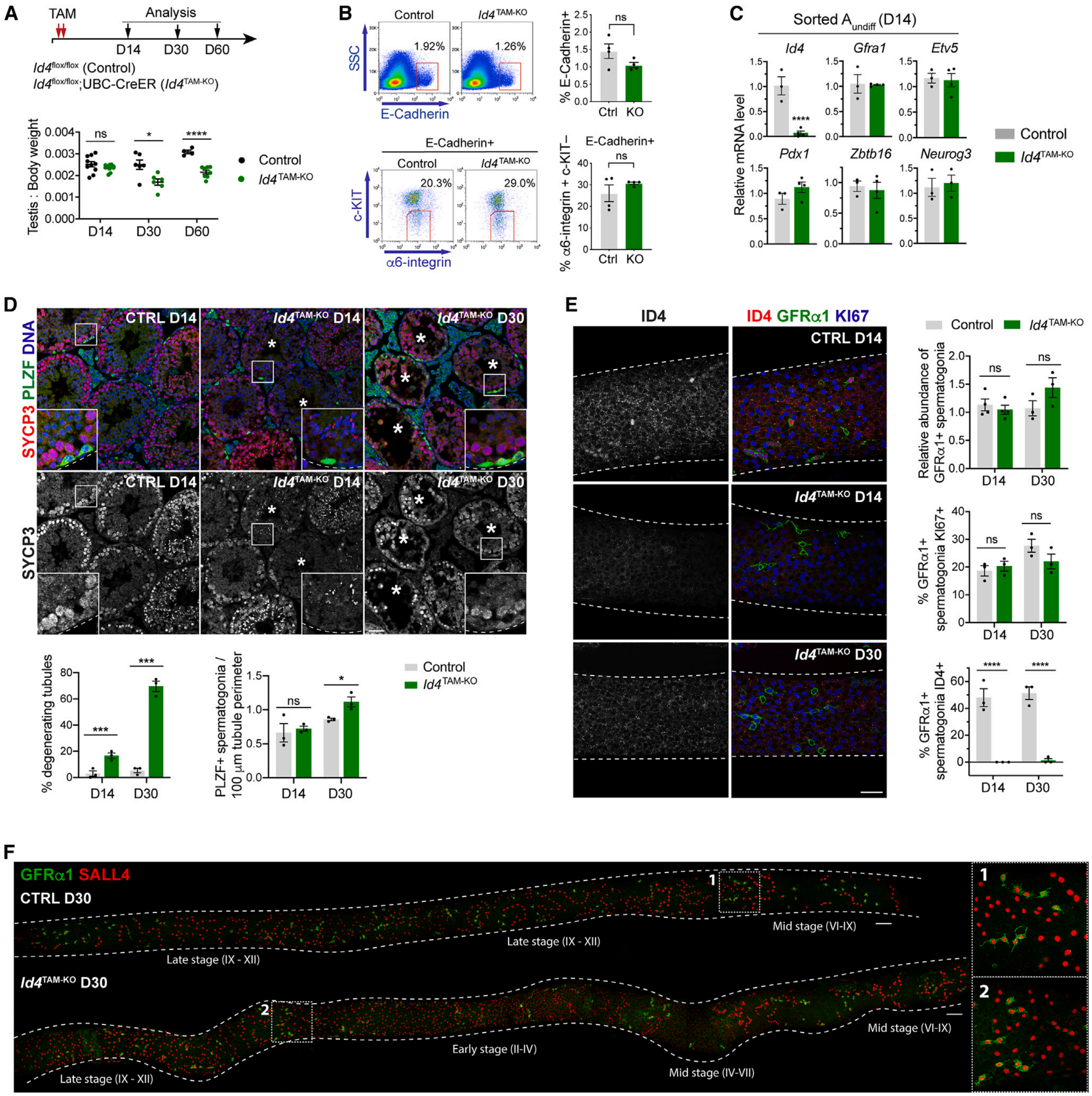

Figure 2. Effects of inducible *Id4* deletion on the adult male germline

(A) *Id4*$^{flox/flox}$ UBC-CreER (*Id4*$^{TAM-KO}$) and Cre-negative littermate control mice were treated with tamoxifen (TAM) and harvested at indicated time points. Lower panel: testis-to-body-weight ratio (n = 4 mice per genotype).

(B) Representative flow cytometry of testis cells from *Id4*$^{TAM-KO}$ and control mice D14 post-TAM. Percentages of cells within gates are indicated. Graphs show percentage of cells E-Cadherin+ (top) and percentage of E-Cadherin+ cells α6-integrin+ c-KIT− (A_{undiff}) (n = 4 mice per genotype).

(C) RT-qPCR of sorted A_{undiff} (E-Cadherin+ α6-integrin+ c-KIT−) from *Id4*$^{TAM-KO}$ and control mice D14 post-TAM (n = 3 controls, n = 4 *Id4*$^{TAM-KO}$ mice).

(D) Representative IF of testis sections D14 and D30 post-TAM. Asterisks: degenerating tubules. Graphs show percentage of tubules degenerating (left) and abundance of PLZF+ spermatogonia (right) (n = 3 mice per genotype and time point, 50 tubule cross-sections scored per animal).

(legend continued on next page)

$Id4^{TAM\text{-}KO}$ and Cre-negative controls (6–8 weeks old) were treated with TAM and analyzed 14, 30, and 60 days (D) later (Figure 2A). Testis-to-body-weight ratios of $Id4^{TAM\text{-}KO}$ mice were unaffected D14 post-TAM but lower at D30 and D60 than controls, suggesting germline depletion (Figure 2A). To study the impact of *Id4* deletion on SSCs, A_{undiff} (E-Cadherin+ α6-integrin+ c-KIT–) were sorted D14 post-TAM (La et al., 2022). The percentage of testis cells E-Cadherin+ and proportion of E-Cadherin+ cells that were undifferentiated (c-KIT–) were unaffected in $Id4^{TAM\text{-}KO}$ mice (Figure 2B). By RT-qPCR, *Id4* expression was substantially reduced in $Id4^{TAM\text{-}KO}$ A_{undiff}, confirming *Id4* deletion (Figure 2C). However, expression of the A_{undiff} marker *Plzf* (*Zbtb16*), SSC-associated genes (*Gfra1*, *Etv5*, *Pdx1*), and the progenitor marker *Ngn3* (*Neurog3*) were unaltered, indicating an intact A_{undiff} pool (Figure 2C).

While IF for germ cell marker VASA suggested a normal seminiferous epithelium in $Id4^{TAM\text{-}KO}$ testis D14 post-TAM (Figure S1A), ~15% of tubules showed depletion of SYCP3+ spermatocytes and degeneration (Figures 2D and S1A). By D30, ~70% of $Id4^{TAM\text{-}KO}$ tubules were degenerating, indicating progressive loss of spermatogenic cells (Figures 2D and S1A). The seminiferous epithelium had partially recovered by D60 although still contained depleted tubules (Figure S1B). Notably, PLZF+ spermatogonia were still present in the basal layer of $Id4^{TAM\text{-}KO}$ degenerating tubules, and abundance was comparable to controls at D14 and slightly increased by D30 post-TAM (Figure 2D). IF of D30 and D60 $Id4^{TAM\text{-}KO}$ testis confirmed ID4 loss in GFP+ A_{undiff} that would normally express *Id4* (Figure S1B) (Best et al., 2014).

By whole-mount IF, the abundance of GFRα1+ spermatogonia and the proportion that were KI67+ were comparable in $Id4^{TAM\text{-}KO}$ and control tubules at D14 and D30 post-TAM, suggesting that SSCs tolerated *Id4* loss and remained mitotically active (Figures 2E and 2F). Loss of ID4 in GFRα1+ cells was confirmed (Figure 2E). RARγ+ progenitors and c-KIT+ differentiating spermatogonia were present as normal in D14 and D30 $Id4^{TAM\text{-}KO}$ tubules (Figure S1C) and seminiferous cycle intact as judged by periodic changes in SALL4+ spermatogonial populations (Figure 2F) (Chan et al., 2017; Tegelenbosch and de Rooij, 1993). Our data indicated that *Id4* deletion resulted in spermatocyte degeneration while SSCs and spermatogonia remained intact. Interestingly, ~80% of the GFRα1+ population in C57BL6 wild-type mice was ID4+, but only ~50% were ID4+ in control mice on a mixed FVBN/CBA/C57BL6 background (Figures 1F and 2E), suggesting that genetic background influences *Id4* expression within the A_{undiff} pool.

Functional redundancy of ID proteins in A_{undiff}

Our analysis indicated that ID4 was dispensable for SSC function. However, *Id4* overexpression blocks SSC differentiation, supporting roles in fate regulation (Helsel et al., 2017). To dissect ID4 function in SSCs, we generated A_{undiff} cultures from $Id4^{TAM\text{-}KO}$ mice and controls D14 after *Id4* ablation (La et al., 2018b). Importantly, $Id4^{TAM\text{-}KO}$ cultures could be established and passaged for at least 6 months (Figures 3A, 3B, and S2A). While control colonies expressed GFRα1 and ID4, cultured A_{undiff} from $Id4^{TAM\text{-}KO}$ mice were GFRα1+ but ID4 negative, confirming *Id4* deletion (Figure 3B). No difference in growth rate between control and *Id4*-deleted cultures was observed (Figure S2B). Further, ID4 loss did not affect the expression of SSC or progenitor-associated genes (*Gfra1*, *Eomes*, *Sox3*, *Neurog3*) or A_{undiff} markers (*Sall4*) (Figure S2C). Therefore, ID4 was not required for the generation or expansion of A_{undiff} cultures.

Given that IDs can have redundant roles (Wang and Baker, 2015), we analyzed *Id* family expression and found that *Id4*-deleted cultures upregulated *Id3* while *Id1* and *Id2* were unaltered (Figure S2C). We confirmed significant ID3 upregulation (~1.5-fold) by western blot (Figure 3C). To assess *Id3* expression following *Id4* deletion *in vivo*, we analyzed $Id4^{TAM\text{-}KO}$ and control testis D30 post-TAM by IF. ID3 levels were increased within GFP+ A_{undiff} following *Id4* ablation (Figure 3D). However, ID3 was not readily detected in spermatocytes of controls or *Id4* knockouts (Figures 3D and S2D), suggesting that ID4 loss is compensated by ID3 in A_{undiff} but not in meiotic cells. Interestingly, ID3 and ID4 expression in wild-type cultured A_{undiff} were regulated by similar pathways including mitogen-activated protein kinase, involved in SSC self-renewal (Hasegawa et al., 2013), supporting redundant roles (Figures 3E and S2E).

ID function is defined by interacting proteins (Roschger and Cabrele, 2017). However, ID-binding partners in A_{undiff} are poorly defined. We therefore characterized ID4-interacting proteins in cultures by immunoprecipitation (IP) and mass spectrometry (Figure 3F; Table S1). As expected, class I bHLH E-proteins including TCF3 (E47) and TCF12

(E) Representative whole-mount IF of tubules at indicated time points and associated graphs from analysis of GFRα1+ spermatogonia (n = 3 or n = 4 mice per genotype and time point).
(F) Representative whole-mount IF of $Id4^{TAM\text{-}KO}$ and control tubules D30 post-TAM. Images taken along tubule length. Stages of seminiferous epithelium regions are indicated. Data are mean ± SEM. Insets show higher magnification details. Scale bars 50 μm (D, E), 100 μm (F). Dashed lines indicate tubule basement membrane or tubule profile. Significance by two-tailed Student's t test ($p > 0.05$ [ns], $*p < 0.05$, $***p < 0.001$, $****p < 0.0001$).
See also Figure S1.

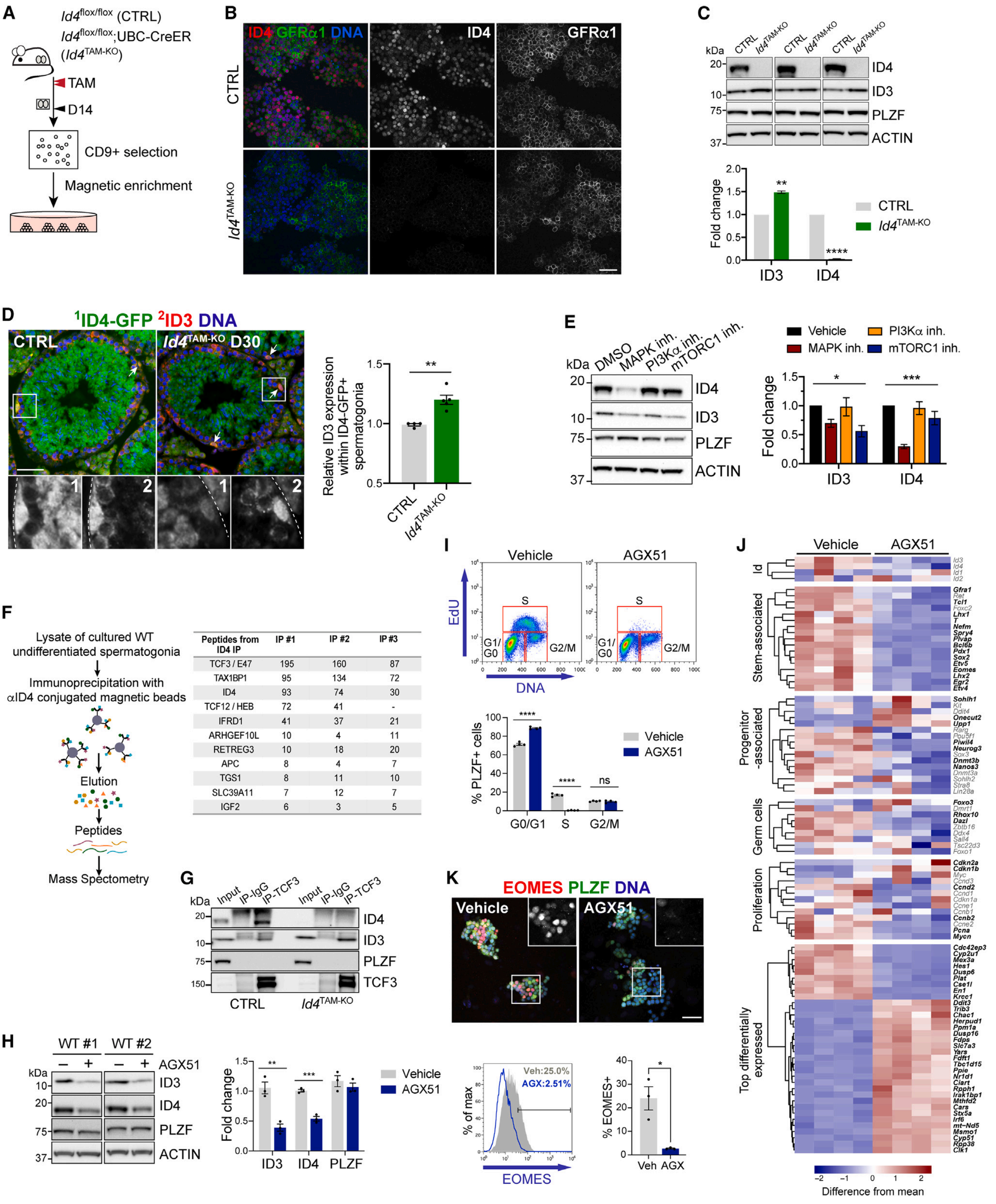

Peptides from ID4 IP	IP #1	IP #2	IP #3
TCF3 / E47	195	160	87
TAX1BP1	95	134	72
ID4	93	74	30
TCF12 / HEB	72	41	-
IFRD1	41	37	21
ARHGEF10L	10	4	11
RETREG3	10	18	20
APC	8	4	7
TGS1	8	11	10
SLC39A11	7	12	7
IGF2	6	3	5

(legend on next page)

(HEB) were identified as ID4 interaction partners (Figure 3F) (Wang and Baker, 2015). Novel interacting proteins included autophagy receptor TAX1BP1 and transcriptional coregulator IFRD1 (Figure 3F) (Iezaki et al., 2016; Kirkin and Rogov, 2019). Gene ontology indicated ID4 associated with factors involved in cell cycle and metabolism (Figure S2F). ID4 associates with TCF3 and TCF12 in mammary epithelium to regulate development and differentiation (Holliday et al., 2021), suggesting similar roles for ID4-TCF3/TCF12 in the male germline. Given that ID3 potentially compensates for ID4 loss, we tested whether ID3 interacted with similar partners. IP of TCF3 from control cultured A_{undiff} confirmed that TCF3 bound both ID4 and ID3 (Figure 3G). Importantly, in *Id4*-deleted cells, interaction between TCF3 and ID3 was robust, consistent with ID3 upregulation and redundant roles of IDs in A_{undiff} (Figure 3G).

ID inhibition disrupts A_{undiff} fate and function

Our data indicated that ID3 and ID4 were functionally redundant in SSCs. *Id1* and *Id2* expression were also detectable, suggesting SSC regulation by all IDs. We therefore treated wild-type A_{undiff} cultures with pan-ID inhibitor AGX51, which binds a conserved cleft in the ID HLH domain and disrupts E-protein interaction (Wojnarowicz et al., 2019, 2021). AGX51 reduced ID3 and ID4, consistent with decreased stability when prevented from binding E-proteins (Figure 3H) (Bounpheng et al., 1999; Wojnarowicz et al., 2019). ID4 reduction was confirmed by IF while GFRα1 was still detected (Figure S2G). AGX51 induced G0/G1 arrest, although it did not trigger substantial apoptosis (Figures 3I and S2H).

RNA sequencing (RNA-seq) of A_{undiff} cultures treated with AGX51 revealed a striking shift in gene expression (Figure 3J; Table S2). While many germ and A_{undiff} markers (*Sall4*, *Ddx4*/*Vasa*, and *Tsc22d3*/*Gilz*) were unaltered, SSC-associated genes were downregulated (e.g., *Eomes*, *Pdx1*, *Lhx1*, *Plvap*, *T*/*Brachyury*, *Gfra1*, and *Spry4*) (La et al., 2018b; Luo et al., 2023). EOMES+ cells were depleted from A_{undiff} cultures by AGX51 (Figure 3K). Progenitor-associated genes were variably affected, with some upregulated (e.g., *Sohlh1*, *Onecut2*, and *Upp1*) and others downregulated (e.g., *Piwil4*, *Neurog3*, and *Nanos3*), suggesting modulation of the differentiation program. Cell cycle inhibitors (*Cdkn2a* and *Cdkn1b*) were upregulated, and genes involved in cell cycle progression (*Ccnd2*, *Ccnb2*, *Pcna*, and *Mycn*) were downregulated, consistent with the role of IDs in proliferation. Expression of *Id* members was not substantially altered. Pathway analysis indicated changes in networks associated with metabolism, DNA repair, cell cycle, and development (Figure S2I).

In summary, we show that within the spermatogonial pool, *Id4* expression is restricted to A_{undiff} and overlaps with SSC marker GFRα1, consistent with the role of GFRα1 as GDNF receptor and *Id4* as a GDNF-induced gene in A_{undiff} (La and Hobbs, 2019; Oatley et al., 2006). However, despite roles of IDs in stem cell maintenance and ability of overexpressed ID4 to block SSC differentiation (Helsel et al., 2017; Lasorella et al., 2014), ID4 loss

Figure 3. Functionally redundant roles of IDs in A_{undiff}
(A) Method for generating A_{undiff} cultures from *Id4*$^{TAM\text{-}KO}$ and control mice D14 post-TAM.
(B) Representative IF of A_{undiff} cultures (passage 6) from A.
(C) Western blot analysis of independently derived A_{undiff} cultures. Graph shows ID3 and ID4 band intensities normalized to actin (n = 3 cultures per genotype from separate mice).
(D) Representative IF of testis sections from control and *Id4*$^{TAM\text{-}KO}$ mice D30 post-TAM (n = 4 mice per genotype). Graph shows ID3 staining intensity in Id4-GFP+ spermatogonia from representative control and *Id4*$^{TAM\text{-}KO}$ mice (minimum 50 tubule cross-sections scored per sample). Dashed lines indicate tubule basement membrane. Arrows: GFP+ ID3+ spermatogonia.
(E) Western blot of cultured A_{undiff} treated for 20 h with inhibitors to indicated pathways. Graph shows ID3 and ID4 band intensity normalized to actin (n = 3 independent cultures from separate mice). Significance by one-way ANOVA.
(F) Identification of ID4 interacting proteins in cultured A_{undiff} using IP and mass spectrometry. Non-specific IgG was used as control. Table includes selected interaction partners from 3 independent IPs alongside numbers of MS2 spectra. Only proteins not identified in control IPs are included.
(G) Confirmation of TCF3-ID interaction in cultured control and *Id4*-deleted A_{undiff} by IP and western blotting.
(H) Western blot of cultured wild-type A_{undiff} treated with AGX51 for 20 h. Graph shows band intensities normalized to actin (n = 3 independent cultures from separate mice).
(I) Cell cycle analysis of wild-type cultured A_{undiff} treated for 24 h with AGX51 by flow cytometry. Graph shows the percentage of PLZF+ cells in different phases (n = 4 independent cultures from separate mice).
(J) RNA-seq of cultured wild-type A_{undiff} treated as in H (n = 4 independent cultures from separate mice). Genes with significant changes in expression are in bold in the heatmap (FDR<0.05, fold change>1.5).
(K) Representative IF of cultured wild-type A_{undiff} treated as in H. Graph shows the percentage of PLZF+ cells EOMES+ (n = 3 independent cultures from separate mice). Insets show higher magnification details. Scale bars 50 μm. Data are mean ± SEM. Significance by two-tailed Student's t test (p > 0.05 [ns], *p < 0.05, **p < 0.01, ***p < 0.001, ****p < 0.0001).
See also Figure S2.

did not disrupt SSC or A_{undiff} function in adults or generation of A_{undiff} cultures. Rather, *Id4* deletion resulted in spermatocyte degeneration, consistent with roles during spermatogenesis and expression at meiotic stages (Hermann et al., 2018). ID3 was upregulated following *Id4* deletion in A_{undiff}, indicating redundant roles in SSCs, supported by the interaction of both ID3 and ID4 with key partner TCF3. Previous analysis of conventional knockout mice indicated roles for *Id4* in SSC maintenance although germline degeneration was most apparent in aged (7–8 months) cohorts (Oatley et al., 2011), suggesting that ID4 loss is reasonably tolerated by SSCs due to compensation by other IDs. The pan-ID inhibitor AGX51 overcomes this redundancy and abolished the SSC gene expression signature of cultured A_{undiff} and induced cell-cycle arrest, validating essential roles for IDs in the maintenance of SSC function.

EXPERIMENTAL PROCEDURES

Mouse models

Id4$^{IRES\text{-}GFP}$ mice have been described previously (Best et al., 2014). See supplemental experimental procedures for details on experimental lines and treatments. Studies were approved by Monash University and Medical Center Animal Ethics Committees.

IF

IF analysis was performed as described (La et al., 2018a, 2022). See supplemental experimental procedures.

Flow cytometry

Methods have been previously described (La et al., 2018a, 2022). See supplemental experimental procedures.

Cell culture

A_{undiff} cultures were generated and treated as described (La et al., 2018b). See supplemental experimental procedures.

IP and western blotting

Methods have been described (La et al., 2018a). See supplemental experimental procedures.

Mass spectrometry

ID4 complexes were immunoprecipitated using rabbit anti-ID4 antibody (clone 82-12, CalBioreagents) (Chan et al., 2017; La et al., 2018a). See supplemental experimental procedures.

RT-qPCR

RNA was extracted using TRIzol LS (Thermo Fisher Scientific) and Direct-zol Kits (Zymo Research). cDNA was synthesized using a Tetro cDNA synthesis kit (Bioline) and analyzed on Mic qPCR Cyclers (Bio Molecular Systems) using Takara Green Premix Ex Taq II (Clontech). Primers are previously described (La et al., 2018b) or from the Harvard Primer Bank (https://pga.mgh.harvard.edu/primerbank/).

RNA-seq

RNA-seq was performed using a multiplex method (Grubman et al., 2021) and analyzed as described (Legrand et al., 2019). See supplemental experimental procedures.

Statistics

Statistical significance was assessed using two-tailed unpaired t tests or one-way ANOVA and GraphPad Prism. p values are indicated as follows: *$p < 0.05$; **$p < 0.01$; ***$p < 0.001$; ****$p < 0.0001$; not significant $p > 0.05$. No statistical method was used to predetermine sample sizes and no specific randomization or blinding methods were used.

RESOURCE AVAILABILITY

Lead contact

Further information and requests for reagents should be directed to and will be fulfilled by the lead contact, Robin Hobbs (robin.hobbs@monash.edu).

Materials availability

Materials can be made available by the corresponding author upon request.

Data and code availability

RNA-seq data are deposited in the Gene Expression Omnibus (GEO) database with accession GEO: GSE254768. Mass spectrometry data have been deposited to the ProteomeXchange Consortium via the PRIDE repository with identifier PRIDE: PXD049346.

ACKNOWLEDGMENTS

We acknowledge Monash Animal Research, FlowCore, Histology, Micro Imaging, Proteomics, Bioinformatics, and MHTP Genomics Platforms. We thank Alex Swarbrick for *Id4*$^{IRES\text{-}GFP}$ mice, Trevor Wilson for RNA-seq analysis, James Bourne for marmoset samples, and Antonella Papa and Julien Legrand for advice. This study used Bioplatforms Australia/NCRIS-enabled infrastructure at the Monash Proteomics and Metabolomics Platform. The Novo Nordisk Foundation Center for Stem Cell Medicine is supported by Novo Nordisk grant NNF21CC0073729. This work was supported by NHMRC Project Grant APP1164019 and ARC Discovery Grant DP220103555 to R.M.H. H.M.L. was supported by an Australian Government Research Training Program Scholarship.

AUTHOR CONTRIBUTIONS

Conceptualization, H.M.L. and R.M.H.; methodology, H.M.L., A.-L.C., A.M.H., F.J.R., R.B.S., and R.M.H.; formal analysis, H.M.L., A.-L.C., A.M.H., B.Y.M.S., R.B.S., and R.M.H.; investigation, H.M.L., A.-L.C., A.M.H., B.Y.M.S., and R.M.H.; writing – original draft, H.M.L. and R.M.H.; writing – review and editing, H.M.L., A.-L.C., R.B.S., and R.M.H.; visualization, H.M.L., A.-L.C., A.M.H., B.Y.M.S., and R.M.H.; supervision, A.-L.C., A.M.H., F.J.R., and R.M.H.; project administration, R.M.H.; funding acquisition, R.M.H.

DECLARATION OF INTERESTS

F.J.R. receives institutional support as a co-investigator and is subcontracted by Peter MacCallum Cancer Centre for an investigator-initiated trial, which receives funding from Sanofi/Regeneron Pharmaceuticals.

SUPPLEMENTAL INFORMATION

Supplemental information can be found online at https://doi.org/10.1016/j.stemcr.2024.08.011.

Received: February 13, 2024
Revised: August 27, 2024
Accepted: August 29, 2024
Published: September 26, 2024

REFERENCES

Best, S.A., Hutt, K.J., Fu, N.Y., Vaillant, F., Liew, S.H., Hartley, L., Scott, C.L., Lindeman, G.J., and Visvader, J.E. (2014). Dual roles for Id4 in the regulation of estrogen signaling in the mammary gland and ovary. Development *141*, 3159–3164. https://doi.org/10.1242/dev.108498.

Bounpheng, M.A., Dimas, J.J., Dodds, S.G., and Christy, B.A. (1999). Degradation of Id proteins by the ubiquitin-proteasome pathway. FASEB J. *13*, 2257–2264.

Chan, A.L., La, H.M., Legrand, J.M.D., Mäkelä, J.A., Eichenlaub, M., De Seram, M., Ramialison, M., and Hobbs, R.M. (2017). Germline Stem Cell Activity Is Sustained by SALL4-Dependent Silencing of Distinct Tumor Suppressor Genes. Stem Cell Rep. *9*, 956–971. https://doi.org/10.1016/j.stemcr.2017.08.001.

Chan, F., Oatley, M.J., Kaucher, A.V., Yang, Q.E., Bieberich, C.J., Shashikant, C.S., and Oatley, J.M. (2014). Functional and molecular features of the Id4+ germline stem cell population in mouse testes. Genes Dev. *28*, 1351–1362. https://doi.org/10.1101/gad.240465.114.

Grubman, A., Choo, X.Y., Chew, G., Ouyang, J.F., Sun, G., Croft, N.P., Rossello, F.J., Simmons, R., Buckberry, S., Landin, D.V., et al. (2021). Transcriptional signature in microglia associated with Abeta plaque phagocytosis. Nat. Commun. *12*, 3015. https://doi.org/10.1038/s41467-021-23111-1.

Hara, K., Nakagawa, T., Enomoto, H., Suzuki, M., Yamamoto, M., Simons, B.D., and Yoshida, S. (2014). Mouse spermatogenic stem cells continually interconvert between equipotent singly isolated and syncytial states. Cell Stem Cell *14*, 658–672. https://doi.org/10.1016/j.stem.2014.01.019.

Hasegawa, K., Namekawa, S.H., and Saga, Y. (2013). MEK/ERK signaling directly and indirectly contributes to the cyclical self-renewal of spermatogonial stem cells. Stem Cell. *31*, 2517–2527. https://doi.org/10.1002/stem.1486.

Helsel, A.R., Yang, Q.E., Oatley, M.J., Lord, T., Sablitzky, F., and Oatley, J.M. (2017). ID4 levels dictate the stem cell state in mouse spermatogonia. Development *144*, 624–634. https://doi.org/10.1242/dev.146928.

Hermann, B.P., Cheng, K., Singh, A., Roa-De La Cruz, L., Mutoji, K.N., Chen, I.C., Gildersleeve, H., Lehle, J.D., Mayo, M., Westernströer, B., et al. (2018). The Mammalian Spermatogenesis Single-Cell Transcriptome, from Spermatogonial Stem Cells to Spermatids. Cell Rep. *25*, 1650–1667.e8. https://doi.org/10.1016/j.celrep.2018.10.026.

Holliday, H., Roden, D., Junankar, S., Wu, S.Z., Baker, L.A., Krisp, C., Chan, C.L., McFarland, A., Skhinas, J.N., Cox, T.R., et al. (2021). Inhibitor of Differentiation 4 (ID4) represses mammary myoepithelial differentiation via inhibition of HEB. iScience *24*, 102072. https://doi.org/10.1016/j.isci.2021.102072.

Iezaki, T., Fukasawa, K., Park, G., Horie, T., Kanayama, T., Ozaki, K., Onishi, Y., Takahata, Y., Nakamura, Y., Takarada, T., et al. (2016). Transcriptional Modulator Ifrd1 Regulates Osteoclast Differentiation through Enhancing the NF-kappaB/NFATc1 Pathway. Mol. Cell Biol. *36*, 2451–2463. https://doi.org/10.1128/MCB.01075-15.

Kirkin, V., and Rogov, V.V. (2019). A Diversity of Selective Autophagy Receptors Determines the Specificity of the Autophagy Pathway. Mol. Cell *76*, 268–285. https://doi.org/10.1016/j.molcel.2019.09.005.

Kitadate, Y., Jorg, D.J., Tokue, M., Maruyama, A., Ichikawa, R., Tsuchiya, S., Segi-Nishida, E., Nakagawa, T., Uchida, A., Kimura-Yoshida, C., et al. (2019). Competition for Mitogens Regulates Spermatogenic Stem Cell Homeostasis in an Open Niche. Cell Stem Cell *24*, 79–92.e76. https://doi.org/10.1016/j.stem.2018.11.013.

La, H.M., Chan, A.L., Legrand, J.M.D., Rossello, F.J., Gangemi, C.G., Papa, A., Cheng, Q., Morand, E.F., and Hobbs, R.M. (2018a). GILZ-dependent modulation of mTORC1 regulates spermatogonial maintenance. Development *145*, dev165324. https://doi.org/10.1242/dev.165324.

La, H.M., and Hobbs, R.M. (2019). Mechanisms regulating mammalian spermatogenesis and fertility recovery following germ cell depletion. Cell. Mol. Life Sci. *76*, 4071–4102. https://doi.org/10.1007/s00018-019-03201-6.

La, H.M., Liao, J., Legrand, J.M.D., Rossello, F.J., Chan, A.L., Vaghjiani, V., Cain, J.E., Papa, A., Lee, T.L., and Hobbs, R.M. (2022). Distinctive molecular features of regenerative stem cells in the damaged male germline. Nat. Commun. *13*, 2500. https://doi.org/10.1038/s41467-022-30130-z.

La, H.M., Mäkelä, J.A., Chan, A.L., Rossello, F.J., Nefzger, C.M., Legrand, J.M.D., De Seram, M., Polo, J.M., and Hobbs, R.M. (2018b). Identification of dynamic undifferentiated cell states within the male germline. Nat. Commun. *9*, 2819. https://doi.org/10.1038/s41467-018-04827-z.

Lasorella, A., Benezra, R., and Iavarone, A. (2014). The ID proteins: master regulators of cancer stem cells and tumour aggressiveness. Nat. Rev. Cancer *14*, 77–91. https://doi.org/10.1038/nrc3638.

Legrand, J.M.D., Chan, A.L., La, H.M., Rossello, F.J., Änkö, M.L., Fuller-Pace, F.V., and Hobbs, R.M. (2019). DDX5 plays essential transcriptional and post-transcriptional roles in the maintenance and function of spermatogonia. Nat. Commun. *10*, 2278. https://doi.org/10.1038/s41467-019-09972-7.

Luo, Y., Yamada, M., N'Tumba-Byn, T., Asif, H., Gao, M., Hu, Y., Marangoni, P., Liu, Y., Evans, T., Rafii, S., et al. (2023). SPRY4-dependent ERK negative feedback demarcates functional adult stem cells in the male mouse germline. Biol. Reprod. *109*, 533–551. https://doi.org/10.1093/biolre/ioad089.

Lyden, D., Young, A.Z., Zagzag, D., Yan, W., Gerald, W., O'Reilly, R., Bader, B.L., Hynes, R.O., Zhuang, Y., Manova, K., and Benezra, R. (1999). Id1 and Id3 are required for neurogenesis, angiogenesis and vascularization of tumour xenografts. Nature *401*, 670–677. https://doi.org/10.1038/44334.

Nakagawa, T., Jörg, D.J., Watanabe, H., Mizuno, S., Han, S., Ikeda, T., Omatsu, Y., Nishimura, K., Fujita, M., Takahashi, S., et al. (2021). A multistate stem cell dynamics maintains homeostasis in mouse spermatogenesis. Cell Rep. *37*, 109875. https://doi.org/10.1016/j.celrep.2021.109875.

Nakagawa, T., Sharma, M., Nabeshima, Y.I., Braun, R.E., and Yoshida, S. (2010). Functional hierarchy and reversibility within the murine spermatogenic stem cell compartment. Science *328*, 62–67. https://doi.org/10.1126/science.1182868.

Oatley, J.M., Avarbock, M.R., Telaranta, A.I., Fearon, D.T., and Brinster, R.L. (2006). Identifying genes important for spermatogonial stem cell self-renewal and survival. Proc. Natl. Acad. Sci. USA *103*, 9524–9529. https://doi.org/10.1073/pnas.0603332103.

Oatley, M.J., Kaucher, A.V., Racicot, K.E., and Oatley, J.M. (2011). Inhibitor of DNA binding 4 is expressed selectively by single spermatogonia in the male germline and regulates the self-renewal of spermatogonial stem cells in mice. Biol. Reprod. *85*, 347–356. https://doi.org/10.1095/biolreprod.111.091330.

Roschger, C., and Cabrele, C. (2017). The Id-protein family in developmental and cancer-associated pathways. Cell Commun. Signal. *15*, 7. https://doi.org/10.1186/s12964-016-0161-y.

Sharma, M., Srivastava, A., Fairfield, H.E., Bergstrom, D., Flynn, W.F., and Braun, R.E. (2019). Identification of EOMES-expressing spermatogonial stem cells and their regulation by PLZF. Elife *8*, e43352. https://doi.org/10.7554/eLife.43352.

Tegelenbosch, R.A., and de Rooij, D.G. (1993). A quantitative study of spermatogonial multiplication and stem cell renewal in the C3H/101 F1 hybrid mouse. Mutat. Res. *290*, 193–200. https://doi.org/10.1016/0027-5107(93)90159-d.

Wang, L.H., and Baker, N.E. (2015). E Proteins and ID Proteins: Helix-Loop-Helix Partners in Development and Disease. Dev. Cell *35*, 269–280. https://doi.org/10.1016/j.devcel.2015.10.019.

Wojnarowicz, P.M., Escolano, M.G., Huang, Y.H., Desai, B., Chin, Y., Shah, R., Xu, S., Yadav, S., Yaklichkin, S., Ouerfelli, O., et al. (2021). Anti-tumor effects of an ID antagonist with no observed acquired resistance. NPJ Breast Cancer *7*, 58. https://doi.org/10.1038/s41523-021-00266-0.

Wojnarowicz, P.M., Lima, E.S.R., Ohnaka, M., Lee, S.B., Chin, Y., Kulukian, A., Chang, S.H., Desai, B., Garcia Escolano, M., Shah, R., et al. (2019). A Small-Molecule Pan-Id Antagonist Inhibits Pathologic Ocular Neovascularization. Cell Rep. *29*, 62–75.e67. https://doi.org/10.1016/j.celrep.2019.08.073.

Yoshida, S. (2019). Heterogeneous, dynamic, and stochastic nature of mammalian spermatogenic stem cells. Curr. Top. Dev. Biol. *135*, 245–285. https://doi.org/10.1016/bs.ctdb.2019.04.008.

Stem Cell Reports, Volume *19*

Supplemental Information

Functionally redundant roles of ID family proteins in spermatogonial stem cells

Hue M. La, Ai-Leen Chan, Ashlee M. Hutchinson, Bianka Y.M. Su, Fernando J. Rossello, Ralf B. Schittenhelm, and Robin M. Hobbs

SUPPLEMENTAL INFORMATION

Functionally Redundant Roles of ID Family Proteins in Spermatogonial Stem Cells

Hue M. La, Ai-Leen Chan, Ashlee M. Hutchinson, Bianka Y.M. Su, Fernando J. Rossello, Ralf B. Schittenhelm and Robin M. Hobbs

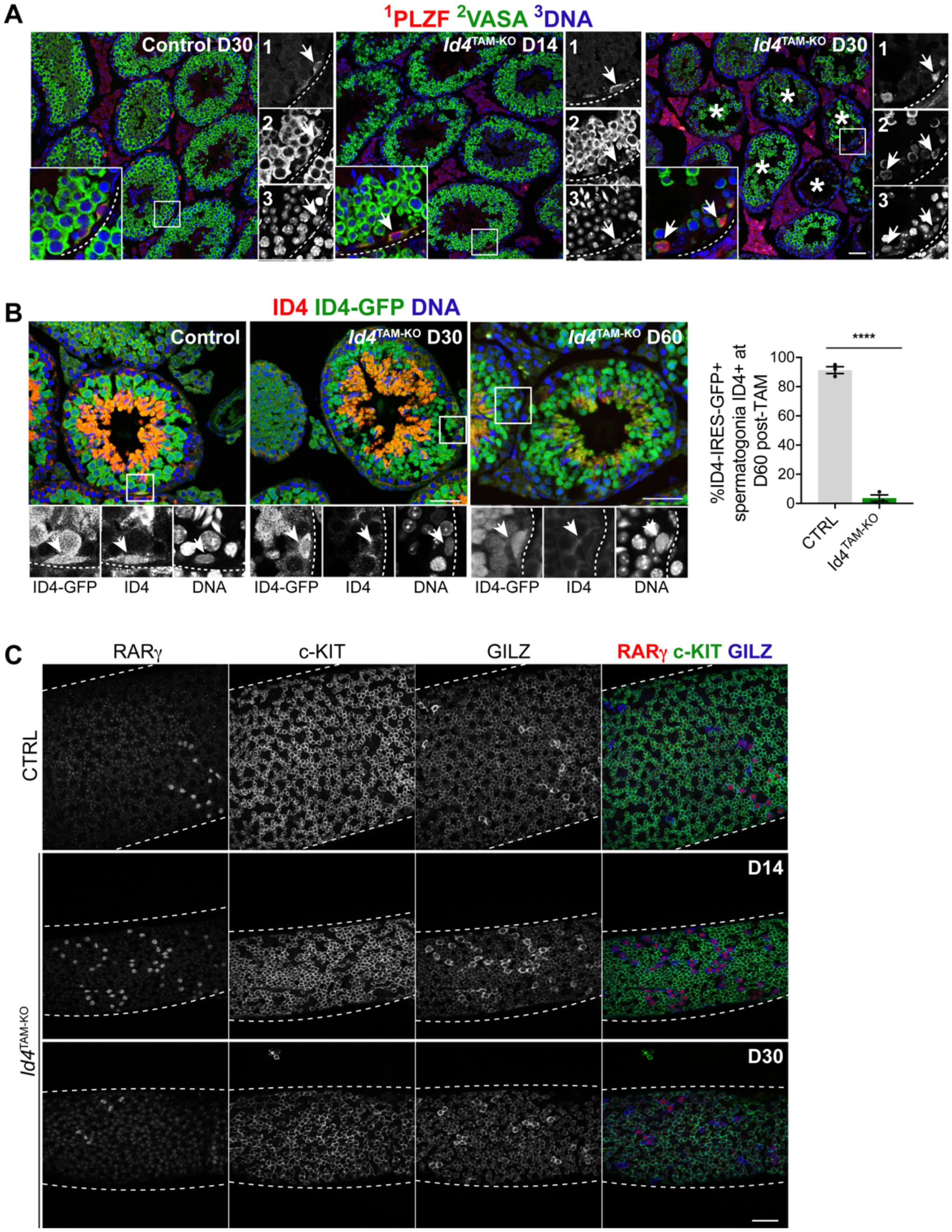

Figure S1. Effects of *Id4* deletion on the adult male germline, related to Figure 2

(A, B) Representative IF of testis sections from *Id4*$^{flox/flox}$ UBC-CreER (*Id4*$^{TAM-KO}$) and Cre-negative control mice treated with TAM and harvested at the indicated time points. Arrows indicate PLZF+ (A) and Id4-GFP+ (B) spermatogonia. Insets show higher magnification details of indicated areas. Asterisks: degenerating tubules. Graph in B shows percentage of GFP+ spermatogonia that are ID4+ D60 post-TAM as an indicator of *Id4* deletion. Data are mean±SEM (*n*=3 mice per genotype).

(C) Representative wholemount IF of seminiferous tubules from *Id4*$^{TAM-KO}$ and control adult mice post-TAM.

Scale bars, 50 µm. Dashed lines indicate tubule basement membrane or tubule profile. Significance by two-tailed Student's t-test (*****P*<0.0001).

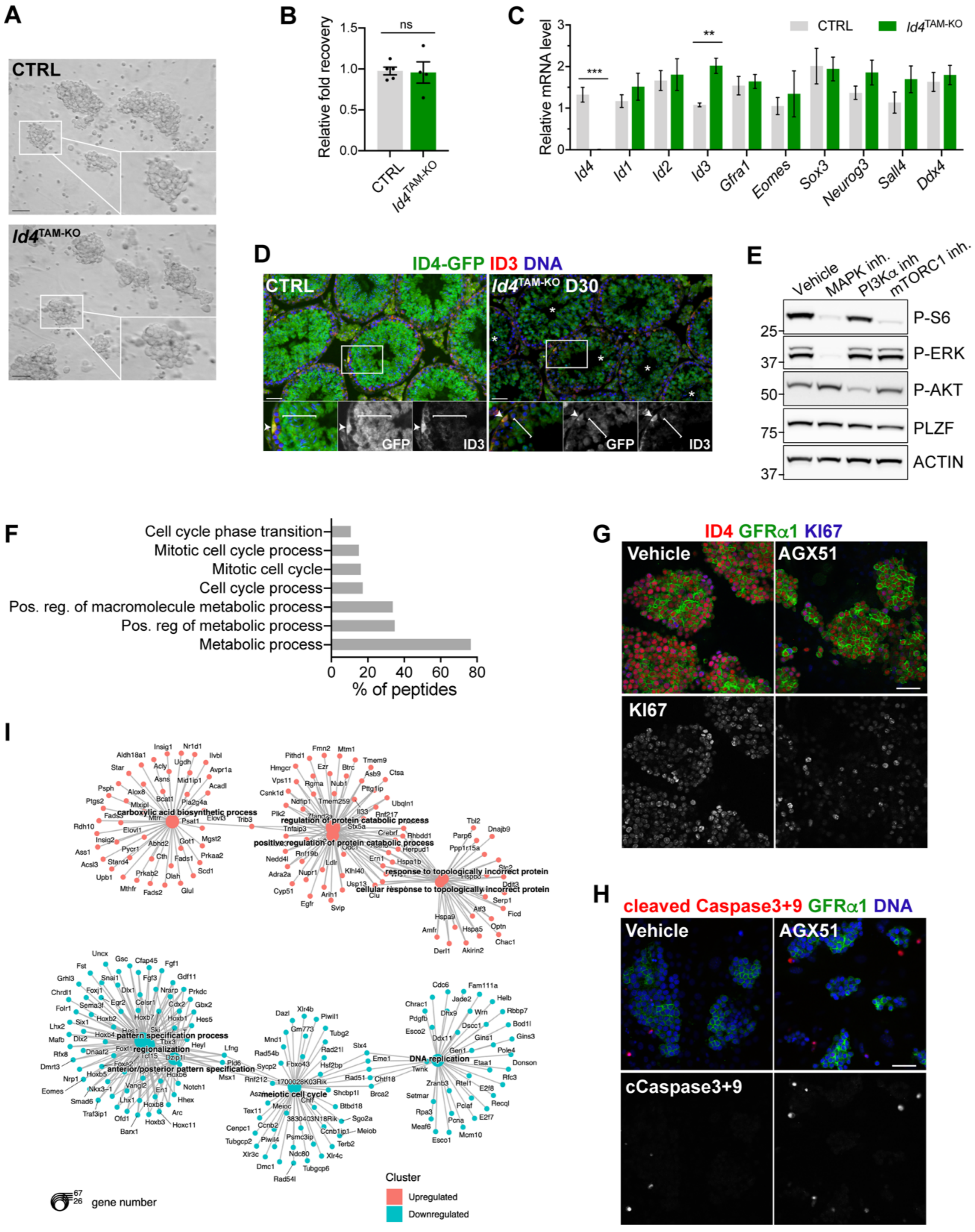

Figure S2. Disruption of *Id* family function in A$_{\mathrm{undiff}}$, related to Figure 3

(A) Phase-contrast light microscope images of A$_{\mathrm{undiff}}$ cultures generated from *Id4*$^{\mathrm{TAM\text{-}KO}}$ and control mice D14 after TAM. Insets show higher magnification details of colonies.

(B) Cultures of A$_{\mathrm{undiff}}$ from A were plated on 12-well plates (0.25x10^{6} cells/well) and expanded for 2 weeks prior to harvest and counting. Graph indicates relative fold cell recovery (*n*=5 control and *n*=4 *Id4*$^{\mathrm{TAM\text{-}KO}}$ independent cultures from separate mice).

(C) RT-qPCR analysis of A$_{\mathrm{undiff}}$ cultures from A. Expression levels are corrected to b-actin and normalized to control sample. Significant changes in expression are indicated.

(D) Representative IF of testis sections from control and *Id4*$^{TAM-KO}$ mice D30 post-TAM (n=4 mice per genotype). Insets show higher magnification details. Asterisks indicate tubules with spermatocyte depletion. Brackets in insets indicate spermatocyte and spermatid populations. Arrows: GFP+ ID3+ spermatogonia. Note that GFP expression from the *Id4*$^{IRES-GFP}$ reporter is maintained following deletion of floxed *Id4* exons although levels are reduced compared to the unrecombined knock-in allele.
(E) Representative western blot of cultured wildtype A_{undiff} treated for 3h with inhibitors to the indicated signalling pathways (*n*=3 independent cultures from separate mice).
(F) Gene ontology analysis of ID4 interacting proteins identified from IP and mass spectrometry analysis of Fig. 3F.
(G, H) Representative IF analysis of wildtype A_{undiff} cultures treated with vehicle or pan-ID inhibitor AGX51 for 20h. Grayscale images of indicated channels are shown.
(I) Pathway analysis of differentially expressed genes identified from RNA-seq of wildtype A_{undiff} cultures treated with vehicle or AGX51 as in Fig. 3J.
Mean values ± SEM are shown in B and C. Scale bars, 50µm. Significance by two-tailed Student's t-test ($P>0.05$ (ns), $^{**}P<0.01$, $^{***}P<0.001$).

Table S1. Identification of ID4 interacting proteins in cultured A_{undiff}, related to Figure 3 (Excel file).

Proteins interacting with ID4 in independent cultures of wildtype A_{undiff} were identified by ID4 IP and high-resolution mass spectrometry analysis as in Figure 3F. The table includes interaction partners identified in at least 2 out of 3 IPs versus non-specific IgG controls. Numbers of MS2 spectra are indicated. Proteins detected in respective IgG control IPs were excluded (-).

Table S2. Analysis of gene expression in cultured A_{undiff} following ID inhibition, related to Figure 3 (Excel file).

Cultured A_{undiff} were treated with vehicle or pan-ID inhibitor AGX51 for 20h and then analysed by RNA-Seq (*n*=4 independent cultures from separate mice). Table shows differentially expressed genes (FDR <0.05 and fold change >1.5).

SUPPLEMENTAL EXPERIMENTAL PROCEDURES

Mouse models and treatment

Wildtype adult mice were 6-8 weeks old and C57BL/6J background. *Id4*$^{\text{IRES-GFP}}$ mice were maintained on a mixed FVBN/CBA/C57BL6 background and are described elsewhere (Best et al., 2014). Mice homozygous for the *Id4*$^{\text{IRES-GFP}}$ allele were used for GFP expression analysis. *Id4*$^{\text{TAM-KO}}$ mice were generated by crossing *Id4*$^{\text{IRES-GFP}}$ mice with the UBC-CreER strain (Jackson Laboratories) (Chan et al., 2017; Ruzankina et al., 2007). The IRES-GFP reporter is retained following Cre-dependent deletion of floxed *Id4* exons (Best et al., 2014). Homozygous *Id4*$^{\text{IRES-GFP}}$; hemizygous UBC-CreER mice were used as experimental mice and homozygous littermate *Id4*$^{\text{IRES-GFP}}$ mice without UBC-CreER as controls. To induce *Id4* deletion, 6–8-week-old adult *Id4*$^{\text{TAM-KO}}$ mice were injected with 2 mg TAM (Sigma) daily for 2 consecutive days as described (Chan *et al.*, 2017). Animal studies were performed in accordance with the Australian Code of Practice for the Care and Use of Animals for Scientific Purposes. Experiments were subject to approval by Monash University and Medical Centre Animal Ethics Committees.

Immunofluorescence

For sections, testes were fixed with 4% paraformaldehyde (PFA) in phosphate-buffered saline (PBS) overnight at 4^0C, cryoprotected with 30% sucrose and embedded in OCT (Tissue-Tek) for sectioning. Sections were blocked in 10% FBS (GE Healthcare) with 2% bovine serum albumin (BSA) (Sigma) in PBS, then incubated at 4^0C overnight with primary antibodies in blocking solution. After PBS washes, sections were incubated with Alexa Fluor-conjugated secondary antibodies raised in Donkey (Thermo) and DAPI. For wholemount analysis, testes were detunicated, seminiferous tubules teased apart and rinsed in PBS, then fixed in 4% PFA for 5 hours

at 4^0C. Fixed tubules were washed in PBS prior to blocking in 0.3% Triton X-100 in PBS (PBSX) supplemented with 10% FBS and 2% BSA. Tubules were incubated with primary antibodies in PBSX with 1% BSA at 4^0C overnight. Samples were washed in PBSX, and primary antibodies detected as above. For IF analysis of cultured A_{undiff}, cells grown on Lab-Tek Chamber slides were fixed in 4% PFA and processed as described (Chan *et al.*, 2017). Slides were mounted in Vectashield mounting medium (Vector Labs) and imaged with Zeiss LSM780 FCS and Nikon C1 confocal microscopes at the Monash Micro Imaging (MMI) platform. ImageJ was used for image processing. Whole-mount tubules were assigned to different stages of the seminiferous epithelium cycle based on abundance and morphology of differentiating and undifferentiated spermatogonial populations as described previously (Chan *et al.*, 2017). Primary antibodies were as follows: rabbit anti-ID4 (clone 82-12, 1:2000) and rabbit anti-ID3 (clone 6-1, 1:2000) (CalBioreagents); chicken anti-GFP (ab13970, 1:5000), rabbit anti-SALL4 (ab29112, 1:2000) and rabbit anti-SYCP3 (ab15093, 1:500) (Abcam); goat anti-PLZF (AF2944, 1:500), anti-GFRα1 (AF560, 1:250), anti-SOX3 (AF2569, 1:250), anti-c-KIT (AF1356, 1:250), anti-E-cadherin (AF748, 1:250) and rabbit anti-EOMES (clone 1219A, 1:1000) (R&D Systems); rat anti-KI67 (clone SolA15, 1:250) and anti-GILZ (clone CFMKG15, 1:1000) (Thermo); rabbit anti-VASA (clone D10C5, 1:500), anti-RARγ (clone D3A4, 1:500), anti-cleaved caspase 3 (#9579, 1:500), and anti-cleaved caspase 9 (#9509, 1:250) (Cell Signaling Technology).

Flow cytometry

Single cell suspensions were generated from testis by digestion with type II collagenase (Sigma) (La et al., 2018a). Cells were stained for 25 minutes on ice with antibodies in phosphate-buffered saline (PBS) with 2% fetal bovine serum (FBS). Antibodies were as follows: PE anti-E-cadherin

clone DECMA-1 (1:250), APC anti-c-KIT clone 2B8 (1:500), PE-Cy7 anti-CD49f (integrin α6) clone GoH3 (1:500) (Thermo and BioLegend). DAPI was used for live/dead cell discrimination. Cell cycle analysis was performed with a Click-iT EdU Pacific Blue Flow Cytometry Kit (Thermo) according to manufacturer's instructions and 3-hour 10μM EdU treatment (Legrand et al., 2019). Analysis of fixed and permeabilised A_{undiff} for PLZF and EOMES is previously described (La et al., 2018b). Cells were sorted and analysed at Monash FlowCore using an Influx Cell Sorter and LSR Fortessa X-20 (BD Biosciences). Data were processed with FlowJo software.

Cell culture and treatment

A_{undiff} cultures were generated and maintained on mitotically-inactivated mouse embryonic fibroblasts (MEF) in StemPro-34 media supplemented with 10ng/ml GDNF, 10ng/ml bFGF, 20ng/ml EGF, 25μg/ml insulin and other additives as described (La *et al.*, 2018a). To establish cultures, A_{undiff} were enriched from testis cell suspensions using biotinylated anti-CD9 antibody clone MZ3 (BioLegend, 1:400) and EasySep Biotin Positive Selection kits (Stem Cell Technologies). For inhibitor treatment, adherent feeder cells were depleted from harvested cells on tissue culture plates for 1-2 hours and non-adherent spermatogonia collected and plated onto 12-well plates coated with Geltrex matrix (Thermo) as detailed (La et al., 2022). Inhibitors (MedChemExpress and Selleckchem) were dissolved in DMSO and diluted in supplemented StemPro-34 media to the following concentrations: 5μM PD0325901 (MAPK inhibitor), 1μM alpelisib (PI3Kα), 20nM rapamycin (mTORC1) and 80μM AGX51 (pan-ID). Media for inhibitor studies contained GDNF, bFGF, EGF, insulin and other supplements as above.

Immunoprecipitation and western blotting

Immunoprecipitation and western blotting were performed as described (La *et al.*, 2018a; La *et al.*, 2022). The following antibodies were used for western blotting: rabbit anti-ID4 (clone 82-12, 1:1000) and anti-ID3 (clone 6-1, 1:1000) (CalBioreagents); rabbit anti-TCF3 (PA5-78190, 1:1000) (Thermo); hamster anti-PLZF (clone 9E12) (Hobbs et al., 2010); rabbit anti-phospho-RPS6 (Ser235/236) (clone D57.2.2E, 1:2000), anti-phospho-p44/42 MAPK (Thr202/Tyr204) (clone D13.14.4E, 1:2000) and anti-phospho-AKT (Ser473) (D9E, 1:2000) (Cell Signaling Technology); and mouse anti-β-ACTIN (Sigma, 1:2000). Band intensity was quantified using ImageJ. Immunoprecipitation with rabbit anti-TCF3 antibody (PA5-78190, Thermo) was performed as detailed below.

Mass spectrometry

ID4 complexes were immunoprecipitated from wildtype cultured A_{undiff} lysate with Dynabeads coupled with rabbit anti-ID4 antibody (clone 82-12, CalBioreagents) using a Dynabeads Antibody Coupling Kit (Thermo Fisher) as described (La *et al.*, 2018a; Legrand *et al.*, 2019). Dynabeads coupled to non-specific rabbit IgG were used as control. Proteins were eluted in 0.2 M glycine pH 2.5 and analysed by the Monash Proteomics & Metabolomics Platform. Immunoprecipitated proteins were reduced with 10 mM TCEP (Thermo), alkylated with 40 mM chloroacetamide (Sigma Aldrich), and digested with sequencing grade trypsin (Promega). Samples were acidified with 1% formic acid (FA) and purified using OMIX C18 Mini-Bed tips (Agilent). Tryptic peptides were separated using a Dionex UltiMate 3000 RSLCnano system and analysed with an Orbitrap Fusion Tribrid mass spectrometer (Thermo). Raw data files were analysed with the Byonic software suite (ProteinMetrics) using a mouse protein sequence database (Uniprot/SwissProt).

RNA-Sequencing

Quality of RNA extracted from A_{undiff} as above was assessed using a Bioanalyzer and RNA quantity by Qubit. RNA-Seq was performed using a custom in-house multiplex method (Grubman et al., 2021). Index was added during initial pA priming and pooled samples amplified using a template switching oligo. P5 was added by tagmentation by Nextera transposase and PCR. Pooled libraries were sequenced on a NextSeq2000 by Monash Health Translation Precinct (MHTP) Medical Genomics Facility. Data were demultiplexed and processed by the Monash Bioinformatics Platform using the RNAsik pipeline. Raw counts files were uploaded to the Degust Webapp (http://degust.erc.monash.edu), which uses limma-voom for statistical analysis. Cut-off for differentially expressed genes was false discovery rate (FDR) <0.05 and fold change >1.5. ComplexHeatmap and clusterProfiler tools (Bioconductor) were used to generate heatmaps and for pathway analysis (Wu et al., 2021).

SUPPLEMENTAL REFERENCES

Hobbs, R.M., Seandel, M., Falciatori, I., Rafii, S., and Pandolfi, P.P. (2010). Plzf regulates germline progenitor self-renewal by opposing mTORC1. Cell *142*, 468-479.

Ruzankina, Y., Pinzon-Guzman, C., Asare, A., Ong, T., Pontano, L., Cotsarelis, G., Zediak, V.P., Velez, M., Bhandoola, A., and Brown, E.J. (2007). Deletion of the developmentally essential gene ATR in adult mice leads to age-related phenotypes and stem cell loss. Cell Stem Cell *1*, 113-126.

Wu, T., Hu, E., Xu, S., Chen, M., Guo, P., Dai, Z., Feng, T., Zhou, L., Tang, W., Zhan, L., et al. (2021). clusterProfiler 4.0: A universal enrichment tool for interpreting omics data. Innovation (Camb) *2*, 100141.
